# Characterization of main pulmonary artery and valve annulus region of piglets using echocardiography, uniaxial tensile testing, and a novel non-destructive technique

**DOI:** 10.3389/fcvm.2022.884116

**Published:** 2022-08-26

**Authors:** David W. Sutherland, Aisling McEleney, Matheus de Almeida, Masaki Kajimoto, Giselle Ventura, Brett C. Isenberg, Michael A. Portman, Scott E. Stapleton, Corin Williams

**Affiliations:** ^1^Bioengineering Division, The Charles Stark Draper Laboratory, Inc., Cambridge, MA, United States; ^2^Seattle Children’s Research Institute, Seattle Children’s Hospital, Seattle, WA, United States; ^3^Department of Mechanical Engineering, University of Massachusetts, Lowell, MA, United States

**Keywords:** cardiac biomechanics, uniaxial tensile test, radial stress, pediatric, pulmonary artery, pulmonary valve, tissue stiffness, animal model

## Abstract

Characterization of cardiovascular tissue geometry and mechanical properties of large animal models is essential when developing cardiovascular devices such as heart valve replacements. These datasets are especially critical when designing devices for pediatric patient populations, as there is often limited data for guidance. Here, we present a previously unavailable dataset capturing anatomical measurements and mechanical properties of juvenile Yorkshire (YO) and Yucatan (YU) porcine main pulmonary artery (PA) and pulmonary valve (PV) tissue regions that will inform pediatric heart valve design requirements for preclinical animal studies. In addition, we developed a novel radial balloon catheter-based method to measure tissue stiffness and validated it against a traditional uniaxial tensile testing method. YU piglets, which were significantly lower weight than YO counterparts despite similar age, had smaller PA and PV diameters (7.6–9.9 mm vs. 10.1–12.8 mm). Young’s modulus (stiffness) was measured for the PA and the PV region using both the radial and uniaxial testing methods. There was no significant difference between the two breeds for Young’s modulus measured in the elastic (YU PA 84.7 ± 37.3 kPa, YO PA 79.3 ± 15.7 kPa) and fibrous regimes (YU PA 308.6 ± 59.4 kPa, YO PA 355.7 ± 68.9 kPa) of the stress-strain curves. The two testing techniques also produced similar stiffness measurements for the PA and PV region, although PV data showed greater variation between techniques. Overall, YU and YO piglets had similar PA and PV diameters and tissue stiffness to previously reported infant pediatric patients. These results provide a previously unavailable age-specific juvenile porcine tissue geometry and stiffness dataset critical to the development of pediatric cardiovascular prostheses. Additionally, the data demonstrates the efficacy of a novel balloon catheter-based technique that could be adapted to non-destructively measure tissue stiffness *in situ*.

## Introduction

Valvular heart disease is a major, complex healthcare burden across the globe, comprised of degenerative diseases, rheumatic heart disease, and congenital defects (1). Often, surgical intervention such as valve replacement is required for survival and improved quality of life. Approximately 280,000 replacement heart valves are implanted globally each year, making it one of the most common cardiac surgical procedures (2). However, the majority of these procedures are in adults; valve replacement in children remains challenging due to a lack of suitable options on the market (3). Valve defects comprise 25% of congenital heart defects, occurring in 8,000–13,000 babies born annually in the United States alone, with cases only predicted to increase in the future (4).

There are currently no commercially available valve prostheses that can meet both the small diameter requirements of newborn and young pediatric patients (<15 mm) and address the need to accommodate growth of the pediatric population (5). In order to achieve regulatory approval, a major challenge that must be overcome is demonstrating safety and efficacy of the device in preclinical animal models. Thus, large animals are an essential tool when developing cardiovascular devices such as heart valve prosthetics. An understanding of the anatomical size constraints and mechanical properties of the tissue at the site of implantation is of critical importance for proper device sizing and minimizing damage to the tissue (6). Such data will allow for a more informed design process that should lead to better study outcomes and require fewer animals. These datasets are especially critical when designing implanted devices that impart forces on the surrounding native tissue, such as self-expanding stents, and, in particular, when designing devices for pediatric patients where there is often limited clinical or historical data for guidance. Relying on mechanical property measurements from adult tissues for pediatric device design may result in poor performing devices, as tissues have been shown to stiffen with age (7, 8). As such, juvenile animal models should be considered for pediatric preclinical studies when matching implantation site geometry and mechanical properties is critical for success. However, there is a scarcity of published data on cardiovascular structure dimensions and tissue stiffness in juvenile animal models for testing pediatric cardiovascular devices.

Currently, there are a few common large animal models used to mimic human cardiac anatomy and physiology for cardiovascular device implantation studies: ovine, canine, and porcine (9). For the purposes of pediatric heart valve intervention, the animal model should bear resemblance to human infant anatomy and growth rates that allow the study of applicable valve sizes. The ovine model has long been used due to similarities with human cardiac anatomy and physiology (10). Ovine models, particularly when compared to other large animal models, have a slower growth rate which can be desirable for chronic studies of host response. Additionally, the cardiac output of lambs is similar to that of human infants and thus likely to impart similar stresses on the surrounding tissue (11). Ovine models are also of value because they can replicate the rapid calcification events observed in human valve replacement (12, 13). A disadvantage of ovine models is their tendency to undergo ventricular fibrillation when there are slight cardiac interventions (14). Ovine models may also be better suited for modeling of larger pediatric patients and adults given their size. The smallest pediatric heart valve replacements reported in sheep to date were implanted at initial diameters of 12–19 mm (15, 16). This is larger than human neonatal valve sizes, which typically range from 7 to 10 mm (17).

Canine animal models have also been used for research in cardiac intervention. Canine models have similar cardiac electrophysiology to humans (18). Their anatomy is more accessible than other animal models and thus allows for easier imaging. They have also previously demonstrated lower rates of infection, given proper care, after valve implantation (9, 18). However, canines can be difficult to obtain approval for surgical testing compared to other animal models. The Institute of American Care and Use Committee has strict restrictions that should be considered when planning canine surgical studies (9).

Porcine models, particularly mini-swine breeds, offer advantages specific to pediatric device development. They have previously been used to study the pathophysiology of congenital heart defects with decreased pulmonary blood flow and are used to practice congenital cardiac surgery due to their small size and weight (19, 20). Another advantage of porcine models is their rapid growth rate which can expedite the study of pediatric devices designed to adapt to growth. Given that mini-swine breeds can replicate the size and weight ranges of human neonates and premature infants (21), it may be expected that piglet cardiac structures would be of similar size to young pediatric patients, but this data has not yet been reported.

While geometric measurement of cardiac structures *in vivo* may be relatively straightforward, techniques to measure mechanical properties *in situ* remain limited. The mechanical properties of individual patients can be estimated non-invasively by imaging vascular deflection across the cardiac cycle (22–25), but cardiovascular devices that mechanically interact with tissue, such as self-expanding stents, require knowledge of tissue stress response in excess of physiologic ranges (6, 26). Physiological pressures usually reside in the elastic regime but forces from implants can extend into the fibrous regime of the stress-strain curve. Well established *ex vivo* techniques exist, such as uniaxial and biaxial tensile testing (27–31), which can provide mechanical information within and beyond physiologic stress regimes. However, they require excision of the tissue from its native site, which is inherently destructive. To our knowledge, it has not been demonstrated that these methods replicate the stress response of living tissue *in situ.* Currently, there are no reported techniques that are capable of non-destructively obtaining patient-specific mechanical properties of tissue when stress regimes above physiologic ranges are of importance.

There were two main objectives of the work presented here: (1) to develop a previously unavailable dataset primarily capturing diameter and stiffness measurements of juvenile porcine PA and the tissue region containing the PV that will inform pediatric heart valve design requirements for preclinical animal studies, and (2) to develop a novel balloon catheter-based method of measuring mechanical properties of tissue non-destructively at and above physiologic pressures, which can be further developed for use *in situ* to inform patient-specific clinical designs. To achieve these goals, we characterized the main pulmonary artery (PA) and pulmonary valve annulus (PV) regions of male Yorkshire (YO, 44 ± 5.8 days old, 10.9 ± 2.5 kg) and Yucatan (YU, 48 ± 3.7 days old, 6.3 ± 0.7 kg) mini-swine piglets. Tissue dimensions were measured by echocardiography and blood pressure was recorded prior to excising the hearts for *ex vivo* mechanical testing. Tests of the PA and PV-containing tissues were first performed with the balloon catheter-based (“radial”) method, followed by excising the tissue for uniaxial testing. We found that juvenile porcine populations provided appropriate cardiovascular geometries and tissue mechanical properties for modeling pediatric pulmonary implants. Additionally, our results indicated that the novel balloon catheter-based methodology provides reasonably accurate tissue stiffness measurements, and shows promise for continued development toward patient-specific, non-destructive *in situ* measurement.

## Materials and methods

### Animal preparation

Male YO (*N* = 8) and YU (*N* = 4) piglets were obtained from Premier BioSource (Ramona, CA, United States). The pigs were initially sedated with an intramuscular injection of ketamine (30 mg/kg body weight), xylazine (2 mg/kg body weight), and atropine (0.04 mg/kg body weight). Monitors were placed for electrocardiogram, pulse oximetry, and rectal temperature. Pigs were intubated with an endotracheal tube, facilitating mechanical ventilation and general anesthesia under inhaled isoflurane (1–3%) with oxygen (40–60%). External jugular venous and femoral arterial access were obtained for continuous blood pressure monitoring, infusion of maintenance fluids, and blood gas measurement by Radiometer ABL 800 (Radiometer America, Westlake, OH, United States).

### *In vivo* anatomical measurements of pulmonary artery and valve annulus

Under the appropriate anesthesia and mechanical ventilation for pigs, we performed anatomical measurements of the PA and PV. This was done using parasternal long-axis and short-axis view of transthoracic and epicardial echocardiography with an ultrasound machine, Sequoia C256 (Siemens, Munich, Germany) with 3.5–7 MHz probes. The cross-sectional diameters of the PV and PA were measured at mid-systolic and end-diastolic phases respectively. When the cross-sectional areas of PA were not circular, we measured maximum and minimum diameters. These values were then averaged by calculating their sum and dividing by 2. Acknowledging the inaccuracies associated with echocardiography as a method of diameter measurement, diameter measurements were repeated later *ex vivo* for comparison, described below. Following the completion of *in vivo* measurements, animals were euthanized and the whole body perfused with cold phosphate-buffered saline (PBS). The whole heart, including large vessels, was extracted from each animal and rinsed three times with PBS, then placed in a freezer bag labeled with study number, animal identification and date of extraction. Freezer bags were placed directly into the −80°C freezer where they remained until being shipped overnight on dry ice for *ex vivo* tests. Similar freezing procedures described by others have shown minimal to no significant effects on bulk tissue mechanical properties (29).

### Tissue preparation for radial testing

YU and YO piglet hearts were stored at −80°C for 1–2 months before *ex vivo* mechanical testing was ready to be performed. As mentioned above, previous studies have shown that freezing porcine cardiac tissue at this temperature results in no statistically significant changes to mechanical properties for up to 1 year (29). On the day of testing, a single heart was removed from the freezer and fully submerged in a beaker of room temperature water until fully defrosted. The bottom third of the heart was removed with a transverse cut to provide simplified access to the PV region and PA through the right ventricle. The connective tissue between the PA and the surrounding large vessels was removed. The heart was then submerged in PBS to avoid dehydration during testing. The PA and PV region of the heart was tested on the same day using both radial and uniaxial testing techniques. This preparation method was repeated for each heart on its respective testing day.

### Radial testing system

**FIGURE 1:**
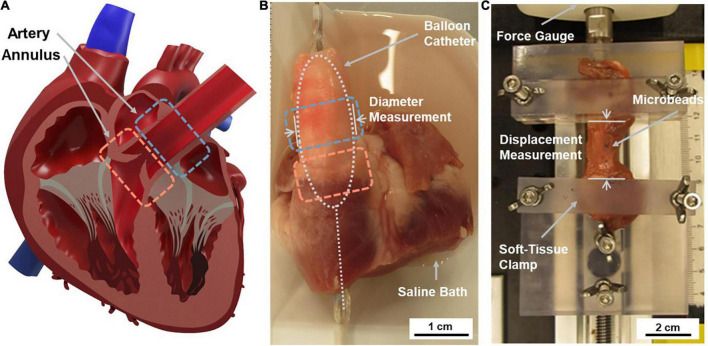
Overview of testing regions of interest and applied tissue testing techniques. **(A)** Tissue samples were sourced from the pulmonary artery (PA) and pulmonary valve (PV) annulus region. **(B)** The radial testing system applied stress to the intact cardiovascular structure using a fluid-filled balloon catheter placed inside the heart. Data was collected using a pressure sensor connected to the balloon catheter system and image analysis of diameter changes. **(C)** The uniaxial testing system applied stress to an isolated circumferential tissue sample at a constant extension rate. Data was collected using force gauge readings and associated displacement measurements.

A TYSHAK II 22 mm non-compliant balloon catheter with a rated burst pressure of > 1.5 atm (NuMed for Children, Inc., Orlando, FL, United States) was used to apply radial pressure from within the main PA and PV tissue region. Applied pressure did not exceed 1.5 atm and the balloon diameter remained under 22 mm for the testing procedures. The balloon catheter was connected to a 20 mL syringe (Plastipak, BD, Franklin Lakes, NJ, United States) mounted in a syringe pump (PHD Ultra, Harvard Apparatus, Holliston, MA, United States) with 1/16” Tygon tubing (Saint Gobain, Courbevoie, France). A barbed tee-junction was used to connect a Luer pressure sensor (Pendo TECH, Princeton, NJ, United States) between the syringe and catheter perpendicular to the flow. Pressure data was recorded using the AcqKnowledge software package and a MP150 data acquisition system (BIOPAC, Goleta, CA, United States). The fluid circuit was purged of air with distilled water. Digital cameras (Dino-Lite Edge AM4115ZTF, Dino-Lite, Torrance, CA and SX40 HS, Canon, Tokyo, Japan) were mounted on perpendicular axes with respect to each other and the balloon catheter guide wire axis to record balloon expansion for PA measurements. Rulers were placed in plane with the measurement axis to provide scale during image analysis. A single digital camera was mounted axially with respect to the balloon catheter guide wire for the PV measurements. The regions of interest for testing are indicated in Figure 1A.

### Radial measurement and testing procedure

The balloon catheter was inserted into the main PA through the right ventricle such that the center of the balloon was aligned ∼1 cm above the PV (Figure 1B). The PA tissue was preconditioned five times before measurements were recorded to reduce tissue hysteresis, eliminate residual stress in the tissue, and ensure that data collection occurred while the tissue was in a repeatable reference state (26, 28). After five cycles of preconditioning, hysteresis curves were shown to overlap, indicating the repeatable state. To precondition, the balloon was inflated at a flowrate of 1 mL/min until reaching 90 mmHg and the total infused volume was recorded. The same volume was then removed to return the tissue to an unstressed state. The recorded volume was infused and removed four additional times. The tissue measurement was performed by inflating the balloon within the PA a final time, at the same flowrate, to 90 mmHg. Diameter values were recorded in place of circumference values because circumference was difficult to measure without interfering with active expansion. Average diameter across 2 axes was measured to account for non-uniform cross-sectional dimensions. Time-stamped pressure data and video were recorded during the test. The balloon catheter was then removed from the heart, and the balloon was inflated without the tissue using the same procedure. Data collected in this step was later used as a baseline measurement in the radial data processing phase described below. The full set of preconditioning and test cycles occurred over the course of 40–50 min while the BIOPAC recorded pressure at a rate of 2 Hz.

After testing, the PA was excised from the heart with a transverse cut ∼0.5 cm above the PV leaflets. The balloon catheter was then placed inside the heart such that the leaflets were positioned at the center of the balloon. Preconditioning and measurement of the PV were performed as described above, and balloon expansion was recorded axially as described in the previous section to record PV diameter changes during testing (Supplementary Figure 1). All YU hearts were measured using the optimized procedure described above. YO hearts, which were tested first, were preconditioned to 50 mmHg and inflated to 150 mmHg. The maximal pressure target was reduced between the YO and YU testing to reduce the risk of plastic deformation during radial testing that could influence the tissue’s response during uniaxial testing.

### Tissue isolation and measurements for uniaxial testing

After radial testing, the tissue was prepared for uniaxial testing. Uniaxial tensile testing is a standard method of mechanical property characterization and was used for comparison against the novel radial testing method. The PA and PV annulus region were tested separately on the uniaxial setup. The PA was isolated from the heart as described in the previous section, and the branches were removed from the top. A vertical cut was made down the posterior side of the artery to unfurl the PA and lay it flat. The length of this cut was recorded as the length of the main PA. From this flat specimen, a rectangular strip with a width of 1 cm was cut in the circumferential direction. The length of this strip of tissue was recorded as the PA circumference (Supplementary Figure 2) and used to calculate diameter. PA sample thickness and width measurements were taken at three equidistant points along the strip using a digital micrometer (Mitutoyo, Kanagawa, Japan) and digital calipers (Mitutoyo, Kanagawa, Japan) respectively. The three values collected at each point were averaged to account for variability in sample dimensions.

The PV region was isolated from the heart by making a transverse incision 1 cm below the previously described incision that separated the PA from the right ventricle. An incision straight through the aorta and right ventricle to the outside of the heart was performed to unfurl the PV region. The excess tissue surrounding the PV region was removed, except for on the ends (∼5 mm) of the tissue strip for clamping. Circumference (length), width, and tissue thickness measurements were taken at three equidistant points along the specimen using calipers and a micrometer, then averaged for each dimension. This step quantified the non-uniformity in specimen dimensions, specifically for the PV region which has variation in thickness along its length.

Rectangular samples were used rather than standard dog-bone shaped samples because specimens were small and non-uniform, making them difficult to cut with precision. Thus, simpler geometry was used to avoid introducing additional non-uniformity between samples. Small tissue dimensions also limited us to only two samples from each piglet heart while maintaining geometric uniformity: one from the PA and one from the PV. The PA was generally shorter than 2 cm in length and curved, resulting in a single 1 cm wide rectangular portion of the tissue that could be isolated for uniaxial testing. Similarly, the area of interest around the PV was constricted by the PA above and at the junction with the right ventricle below.

### Uniaxial tensile testing system

The tissue samples were mounted on the uniaxial testing system between two custom 3D printed tissue clamps. These clamps had surfaces textured with a checked pattern to minimize slippage and were tightened by hand (Figure 1C). The static clamp was attached to a Mark-10 M3-2 Digital Force Gauge (Mark-10, Copiague, NY, United States) and the dynamic clamp was affixed to the carriage of a linear stage actuator (5236A17, McMASTER-CARR, Douglasville, GA, United States). This carriage was actuated toward and away from the force gauge using a NEMA 23 Unipolar Stepper Motor (1477, Pololu Corporation, Las Vegas, NV, United States). The stepper motor’s motion was dictated by an Arduino Uno Rev 3 (Arduino, Somerville, MA, United States), which was programmed with the length of each specimen to calculate the preconditioning and testing sequence. The extension rate of the system (0.06 mm/s) was used to calculate the specimen’s displacement.

### Uniaxial tensile testing procedure

The tissue samples were mounted with approximately 5 mm of tissue held in each clamp and adjusted to remove slack without producing a force reading (Figure 1C). The starting distance between the clamps was then measured and recorded. Three blue microbeads were then placed on the sample: one on each side where the tissue met the clamps to show if slippage occurred, and one marker bead in the center.

Preconditioning cycles were performed five times to 30% strain at a rate of 0.06 mm/s to align with the radial testing system. Preconditioning was repeated after radial testing to address potential changes in the tissue state induced from the isolation process, and additionally served to eliminate any mounting issues (e.g., slippage in clamps) before data acquisition was performed. The strain rate for uniaxial testing was selected to match the strain rate of the radial system. It was important to match strain rates between the testing methods because PA and PV tissues, like many soft biological tissues are viscoelastic, and measured responses in viscoelastic tissues can be affected by strain rate. The sample was stretched until fracture during the test cycle at the same extension rate. The full set of preconditioning and test cycles occurred over the course of approximately 30 min while the force gauge recorded tensile stress values at a rate of 1 Hz. The exposed surface of the tissue was hydrated manually for the duration of testing using a syringe filled with PBS.

All YU hearts were tested using the procedure described above. YO hearts were preconditioned ten times instead of five. Preconditioning cycles were reduced from ten to five between testing the YO and YU specimens in order to shorten the total testing time; we also found that the hysteresis curves overlapped sufficiently after five cycles. Additionally, YO extension rates were 0.6 mm/s rather than 0.06 mm/s for both preconditioning and test cycles. This adjustment to the testing protocol was made after YO data was obtained to better match the uniaxial extension rate to the radial extension rate.

### Radial data processing

The baseline pressure of the balloon was subtracted from the corresponding tissue pressure dataset to isolate the pressure induced by the tissue’s stress response. The initial diameter measurement was recorded as the isolated pressure curve rose above 0 mmHg. Vessel diameters were extracted from the video images using ImageJ (NIH, Bethesda, MD, United States). The internal vessel diameter was estimated for PA samples by subtracting two times the average wall thickness from the measured external vessel diameter. The internal diameter was calculated directly for the PV samples. Diameter measurements were recorded in two perpendicular axes for all samples and averaged. Images were taken from the digital recordings at 10 s increments for the PA data sets and 5 s increments for the PV data sets and aligned with the corresponding pressure measurements. The time increments were reduced for the PV to provide more data points since less volume was needed to reach 90 mmHg pressure. Engineering strain was calculated at each time point using the vessel internal radius (*Eq. 1*) while engineering stress was calculated at each time point using the thin-walled hoop stress equation (*Eq. 2*),


(1) $$\varepsilon_{h}=\frac{r_{i}-r_{o}}{r_{o}}$$

(2) $$\sigma_{h}=\frac{p_{i}*r_{i}}{t_{o}}$$

where *ε_h_* is the engineering strain calculated using the hoop stress equation, *r*_*i*_ is the radius at each time point, and *r*_*o*_ is the initial unstressed radius. *σ_*h*_* is the engineering stress calculated using the hoop stress equation, *p*_*i*_ is the pressure at each time point, *r*_*i*_ is the radius at each time point, and *t*_*o*_ is the circumferential wall thickness.

### Uniaxial tensile data processing

Force gauge readings, *F*_*i*_, were paired to their corresponding displacement values by time stamp. Time values were multiplied by the extension rate (0.06 mm/s) to determine the sample’s displacement at the given point in time. The sample’s cross-sectional area, *A*_*o*_, was calculated by multiplying the initial average width measurement by the initial average thickness measurement. By dividing each force gauge reading by the initial cross-sectional area, axial engineering stress, *σ_*a*_*, was calculated (*Eq. 3*). Similarly, each displacement value was divided by the sample’s initial length, *L*_*o*_, to calculate axial engineering strain, *ε_*a*_* (*Eq. 4)*.


(3) $$\sigma_{a}=\frac{F_{i}}{A_{0}}$$

(4) $$\varepsilon_{a}=\frac{△\,x}{L_{0}}$$

### Calculation of tissue stiffness

Tissue stiffness (Young’s modulus) was estimated using a linear approximation of the elastic and fibrous regions of each sample’s stress-strain curve. Linear fits and residuals were calculated in the NumPy library for Python (Wilmington, DE, United States) using the polyfit method and correlation coefficient function, respectively. Linear fits were calculated over a stress range of 10 kPa for both the elastic and fibrous regions. The stress ranges were selected to provide the highest quality approximation for each tissue type (PA and PV) and stress regime (elastic and fibrous). Fit quality was determined by residual squared values, R^2^, which were averaged across the experimental group. The stress range associated with the highest average R^2^ values was selected as the optimal linear approximations. Both YU and YO breeds were grouped together by tissue type (PA and PV region) to define elastic and fibrous regimes and compare Young’s modulus measurements. For the PA tissue samples, 3–13 kPa and 26–36 kPa were selected for the elastic and fibrous regions, respectively. For the PV tissue samples, 0–5 kPa and 10–20 kPa were selected for the elastic and fibrous regions, respectively. Note that the PV elastic stress range is less than 10 kPa due to the relatively rapid onset of tissue transition compared to the PA tissue.

Additionally, an average stress response curve was produced from the YU uniaxial PA samples in order to compare the porcine data to additional data sets provided in other publications. Due to the large strain variations, a strain-averaged method was used to solve for the average tissue strain for the common applied stress range. The standard deviation of the strain was calculated and is presented as a horizontal error when plotted as a standard stress response curve.

### Curve fit analysis

A curve fitting analysis was also performed on the stress response measurements for each tissue sample to provide an instantaneous Young’s modulus across the elastic, transitional, and fibrous regimes. A non-linear regression analysis was performed using the Curve Fitting Toolkit in MATLAB. The full data sets were used for the radial technique and the uniaxial techniques were truncated at a stress of 60 kPa to align with the available radial technique stress range. A two-term exponential method (*Eq. 5*) was chosen for all data sets and a residual analysis was performed to determine regression error and confirm the two-term exponential method as the most appropriate fit:


(5) $$\varepsilon =a\,e^{b\,\sigma }+c\,e^{d\,\sigma }$$

where *a*, *b*, *c*, and *d* are the output coefficients of the regression analysis, and ε is the instantaneous strain. Instantaneous Young’s modulus values were calculated from the curve fit equations by taking the inverse of the first derivative.

### Statistical analysis

A variety of statistical analyses were performed, as appropriate, using GraphPad Prism software (version 9.3.1). For comparisons of two groups, the Student’s *t*-test was used. A two-way ordinary analysis of variance (ANOVA) with Tukey’s *post hoc* test was used to compare Young’s modulus in the elastic and fibrous regimes as well as compare values measured within each regime by the radial and uniaxial testing methods. For the comparison of Young’s modulus measured by the radial and uniaxial methods as a function of pressure, the multiple unpaired *t*-test with Welch correction and assumption of individual variance for each group was used. Differences were considered statistically significant for *p*-values < 0.05. Bland-Altman analysis, specifically the difference vs. average method (32), was used to assess the limits of agreement between the radial and uniaxial testing methods for determining Young’s modulus in the elastic and fibrous regimes of the three tissues (YU PA, YU PV, and YO PA). Outliers in this analysis were noted but not removed.

## Results

### Measurement of pulmonary artery and pulmonary valve tissue dimensions

Anatomical measurements were collected first from the YO piglets (*N* = 8) and later from the YU (*N* = 4). The YU and YO piglet weights were 6.33 kg ± 0.67 kg and 10.93 kg ± 2.54 kg respectively. Although the ages were similar, YO piglets had significantly higher weights which corresponded with larger anatomical structures. Four piglets of each breed had *in vivo* measurements performed, unless otherwise noted (summarized in Supplementary Table 1).

**FIGURE 2:**
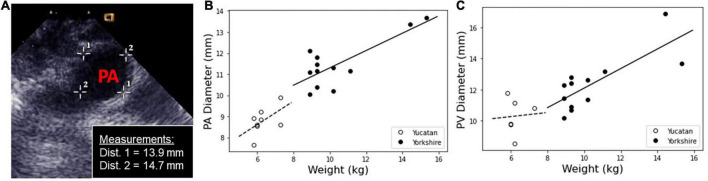
Measurements of PA and PV diameters were taken *in situ* using echocardiogram, and calculated *ex vivo* from the tissue sample circumference. **(A)** The *in vivo* measurements of the PA and PV were measured using a cross-sectional image within the echocardiogram software. A representative image is shown with PA diameter measurements. **(B)** PA and **(C)** PV diameter measurements for Yucatan (YU) and Yorkshire (YO) populations as a function of weight.

PA and PV diameter measurements are shown for YU and YO hearts taken by *in vivo* echocardiogram and *ex vivo* circumference measurements by calipers (Figure 2). YU PA diameters were 8.7 mm ± 0.9 mm by echocardiogram and 8.8 mm ± 0.3 mm by calipers, while YO PA diameters were 10.9 mm ± 0.8 mm and 11.8 mm ± 1.2 mm (*N* = 8), respectively.

YU PV diameters were 8.9 mm ± 1.0 mm by echocardiogram and 10.9 mm ± 0.8 mm by calipers. YO PV diameters were 12.1 mm ± 1.0 mm and 12.5 mm ± 2.1 mm (*N* = 8) respectively. Overall, YO PA and PV diameters were significantly larger than the lower weight YU piglets. By echo measurements, PA and PV diameters were similar for each respective breed. There was generally good agreement between the echo and caliper-based measurements, with the exception of the YU PV diameter values.

In addition, we measured PA wall thickness and PA length *ex vivo* using a micrometer and calipers respectively. The PA thickness was 1.5 mm ± 0.2 mm and 1.5 mm ± 0.3 mm for the YU and YO breeds respectively, while the PA lengths were 15.5 mm ± 1.9 mm and 19.6 mm ± 4.9 mm for the YU and YO breeds respectively.

### Tissue mechanical properties

**FIGURE 3:**
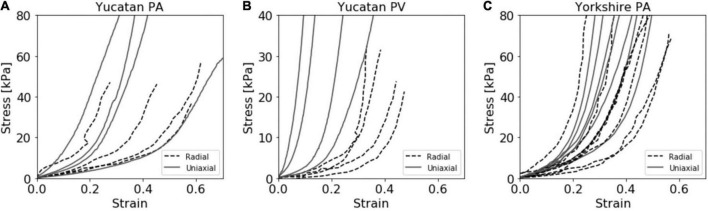
Compiled stress vs. strain curves for both radial and uniaxial tensile testing methods for the **(A)** Yucatan pulmonary artery (PA), **(B)** Yucatan pulmonary valve (PV) region, and **(C)** Yorkshire PA tissue samples.

Experimental stress and strain data is shown for all breeds, tissues, and techniques in Figure 3. The stress response curves show an initial linear response at low stresses indicative of the elastin dominated (elastic) regime. As stress increases, this regime transitions into the stiffer collagen dominated (fibrous) regime, as is typical of cardiovascular tissues (33). These linear regions represent the dominance of one regime over the other and are commonly used to estimate tissue stiffness. The transition region is the non-linear region of the stress-strain curve between the two linear sections, where the elastic region occurs before the transition, and the fibrous region after.

Unlike the PA tissue, a quantifiable amount of pressure was required to lift the PV tissue region and restore it to a cylindrical shape. The internal diameter datasets showed that the horizontal diameter measurement reduced as the vertical axis increased until a discrete point in both curves where a constant relationship was reached and maintained for the remainder of the experiment (Supplementary Figure 3). This re-conformation pressure produced large strain values for the radial YU PV experimental group which were unlikely to be representative of the true stress response. For this experimental group, the r_0_ value was determined to be the point of alignment between the measurement axis and the calculated stress from the re-conformation pressure was removed from the final stress response curve. This point was determined individually for each sample. The adjusted stress value for the YU PV data set was 1.38 ± 0.40 kPa.

**FIGURE 4:**
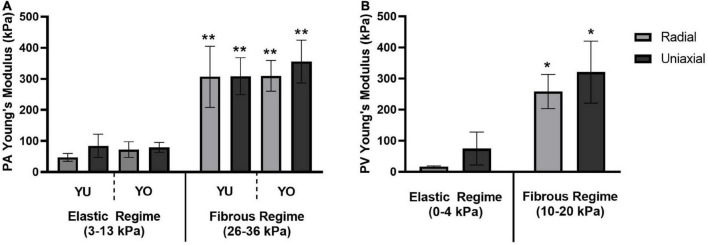
Young’s Modulus for elastic and fibrous regimes. A comparison of Young’s Modulus values determined by the radial and uniaxial testing methods for **(A)** the pulmonary artery of both Yucatan and Yorkshire piglets, and **(B)** the pulmonary valve annulus region of Yucatan piglets. There was no significant difference in Young’s Modulus measurements between testing methods or breeds within a given regime. The Young’s Modulus of the fibrous regime was significantly higher compared to its corresponding elastic regime (* indicates *p* < 0.0005; ** indicates *p* < 0.0001).

Young’s modulus values were estimated by linear regression for the elastic and fibrous regimes of the stress-strain curves (Figure 4). Mean values and standard deviations are reported in Table 1. The elastic and fibrous regimes were estimated to be 3–13 kPa and 26–36 kPa, respectively, for PA tissue samples and 0–4 kPa and 10–20 kPa, respectively, for PV tissue samples. The elastic and fibrous regimes for a given tissue, breed, and measurement technique were significantly different (*p* < 0.0005). There was no significant difference between breed (YU vs. YO) or measurement technique (radial vs. uniaxial) within the respective elastic or fibrous regimes for the PA and PV data sets.

**TABLE 1:** Summary of Young’s modulus values.

			Uniaxial		Radial	
Tissue Type	Tissue Regime	Species	Young’s Modulus (kPa)	R^2^	Young’s Modulus (kPa)	R^2^
Pulmonary Artery	Elastic	Yucatan	84.7 ± 37.3	0.98 ± 0.01	47.3 ± 12.5	0.98 ± 0.01
		Yorkshire	79.3 ± 15.7	0.98 ± 0.01	72.4 ± 25.3	0.97 ± 0.02
	Fibrous	Yucatan	308.6 ± 59.4	1.00 ± 0.00	306.7 ± 98.4	0.99 ± 0.01
		Yorkshire	355.7 ± 68.9	0.99 ± 0.00	309.7 ± 49.5	0.97 ± 0.05
Pulmonary Valve	Elastic	Yucatan	74.7 ± 52.9	0.96 ± 0.02	16.7 ± 2.2	0.91 ± 0.03
	Fibrous	Yucatan	320.9 ± 99.9	0.99 ± 0.00	258.4 ± 54.7	0.96 ± 0.06

### Agreement between radial and uniaxial testing methods

**FIGURE 5:**
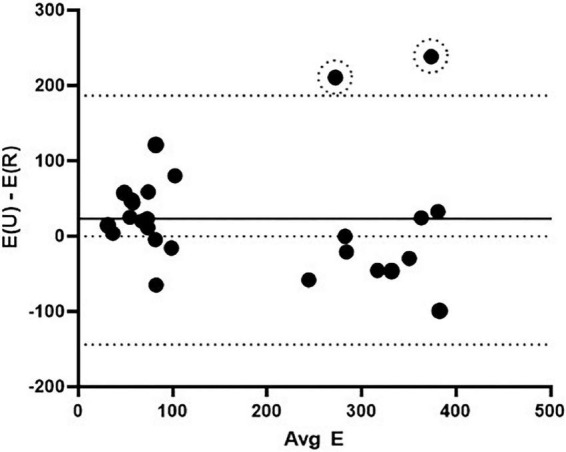
Bland-Altman agreement analysis between the radial and uniaxial methods for determining elastic modulus. The solid middle line indicates the bias and the bounding dashed lines indicate the limits of agreement (± 1.96 SD). Data included are YU PA, YU PV, YO PA elastic modulus in the elastic and fibrous regimes (*N* = 30). Two outliers are noted by dashed circles. E(U) – E(R) = the difference in Young’s modulus values between the uniaxial and radial testing methods, and Avg E = the averaged Young’s modulus values for the two methods for a given stress.

The Bland-Altman method (32) was used to determine agreement between the novel radial technique and the “gold standard” uniaxial tensile testing technique. The difference vs. average plot for all elastic and fibrous regime Young’s modulus measurements (*N* = 30) is shown in Figure 5. The bias was roughly uniform at 21.4 kPa with a standard deviation of 84.4 kPa. There were two outliers from the limits of agreement (YO PA, 9.9 kg piglet and YU PA, 6 kg piglet; both fibrous regime). These outliers were not removed from the analyses shown in Figure 5; however, when they are removed the bias reduces to 6.9 ± 65.9 kPa. Overall, the Bland-Altman method indicated that the agreement between the radial and uniaxial testing methods was within acceptable limits.

### Instantaneous tissue stiffness

**FIGURE 6:**
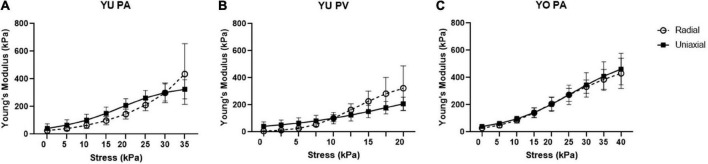
Young’s modulus vs. stress for radial and uniaxial testing methods. Graphs are shown for **(A)** Yucatan piglet pulmonary artery (PA), **(B)** Yucatan piglet pulmonary valve (PV) region, and **(C)** Yorkshire pulmonary artery (PA). There is no significant difference in Young’s modulus measurements for a given stress between the two methods.

The exponential curve fit analysis allowed for the Young’s modulus to be calculated at any point along the experimental stress response curve. In addition, it served to smooth the effects of experimental noise in the data sets. This approach may provide a more accurate assessment of the instantaneous Young’s modulus compared to linear approximations. However, when comparing Young’s modulus values between individual test curves within the same population, the large strain variation limited the ability to plot average Young’s modulus against tissue strain. It was observed that plotting Young’s modulus versus applied stress provided a much better alignment of techniques within our sample population (Figure 6). There was no significant difference between Young’s modulus values determined by radial and uniaxial techniques at any given stress, further indicating good agreement between these methods.

## Discussion

This work represents the first published dataset providing anatomical measurements (PA and PV diameters, PA length, PA wall thickness) and tissue stiffness calculations (Young’s modulus in the physiologic, elastic and super-physiologic, fibrous regimes) for juvenile mini-swine models. We expect this data will be valuable to inform pediatric medical device development, specifically for devices that are designed for implantation in the PA or PV location. We also developed a novel technique to measure tissue stiffness using a balloon catheter-based radial testing system that could be adapted for *in situ* use in future work. Further discussion of these topics and limitations of the study are provided below.

### Juvenile porcine pediatric models

Ovine models have traditionally been used as a preferred animal model for pulmonary valve implants due to their similarity in physiology to humans and accelerated calcification response (12, 34). Indeed, several recent studies have shown promising results with pediatric heart valves implanted in sheep (15, 16, 35). However, pediatric PA tissue was found to be thinner, and more anisotropic compared to ovine PA tissue, and was also significantly stiffer when strained beyond normal physiological conditions (28), although it should be noted that only a single pediatric donor was used. These differences in tissue characteristics could affect performance of devices that impart force on the surrounding tissue, as is common with stents used in heart valve prosthetics. Relatively more data is available characterizing the mechanical properties of adult human cardiovascular tissue (36–38). However, the use of adult data to inform pediatric device design is not ideal, as tissue mechanical properties have been shown to change with age (7, 8). Juvenile swine models have been used for the development and testing of cardiovascular pediatric devices and modeling of congenital heart diseases (21, 39–42), although use is currently limited in the context of pediatric valve design (43). Data from the YU and YO piglets presented here demonstrated similarities to pediatric patients in PA and PV anatomy sizes and tissue stiffness relative to currently available ovine data.

Our data showed that across the two breeds studied, echo measurements of juvenile porcine PA and PV diameters fell between 7.6 and 12.8 mm for piglets weighing between 5.8 kg and 15.3 kg. The measured juvenile porcine diameters roughly align with the range of PV diameters reported for neonates to children a few years of age (5). In addition, our micrometer and caliper measurements of piglet PA wall thickness and PA length were similar to human infants ages 6–8 months (15, 28). It should be noted that the research by Zilberman et al. was performed on healthy tissue, and the anatomy of patients with congenital heart defects, who would require valve prosthetics, may differ. The comparison of PA size and tissue stiffness across the presented juvenile porcine breeds provides information that will potentially help researchers improve pediatric heart valve design and select appropriate preclinical animal models.

**FIGURE 7:**
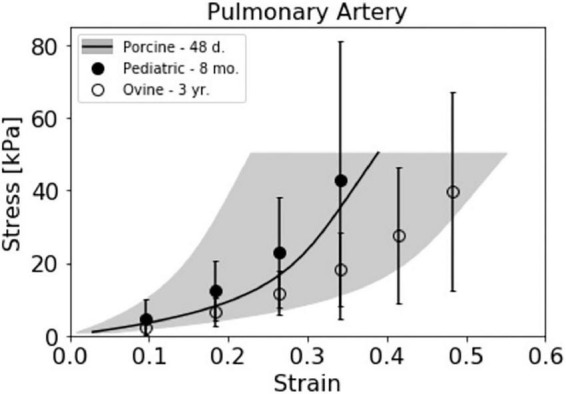
Juvenile porcine pulmonary artery (PA) stress response curve compared with pediatric relevant data sets. The strain averaged Yucatan PA data from the uniaxial testing method (*N* = 4) is presented alongside 8 month old pediatric (*N* = 1) and 3 year old ovine (*N* = 4) stress response curves previously presented by Cabrera et al. (28). Stress response data from Cabrera et al. was estimated from the reported secant modulus averages for the respective species.

The available data on PA and PV tissue characteristics from various species is limited and often presented such that direct comparisons are difficult. Nevertheless, we compiled Table 2 to present measurements of PA and PV anatomy for porcine and ovine models, as well as variations with age, for comparison to human pediatric and adult data. Additionally, estimated stress response curves were calculated from secant modulus data for ovine and pediatric PA circumferential mechanical testing from a published study (28), and plotted against our strain-averaged uniaxial stress response curves (Figure 7). While there is significant biological variation, our piglet PA stress-strain data matches relatively well with their reported human infant PA tissue. Taken together, the anatomical and tissue stiffness measurements of mini-swine align well with human pediatric patients, suggesting that juvenile porcine models could be useful for preclinical pediatric cardiovascular device studies.

**TABLE 2:** Comparison of measurements across species and ages.

Species	Age	Size	PA wall thickness (mm)	PA diameter (mm)	PA length (mm)	PV diameter (mm)	Sample size (# of donors)	References
***Porcine***								
Yucatan, pediatric	48 ± 3.7 days	6.3 ± 0.7 kg	1.5 ± 0.2	8.7 ± 0.9	19.6 ± 4.9	8.9 ± 1.0	*N* = 4	
Yorkshire, pediatric	44 ± 5.8 days	10.9 ± 2.5 kg	1.5 ± 0.3	10.8 ± 0.8	15.5 ± 1.9	12.1 ± 1.0	*N* = 4–8	
Unspecified, adult			2.53 ± 0.47		20.0 ± 3.6		*N* = 10	Matthews (50)
Swiss large white, adult		50–60 kg		21.28 ± 2.06	52.13 ± 5.04	25.97 ± 1.37	*N* = 8	Lipiski (51)
Swiss large white, adult		100–110 kg		26.86 ± 4.08	66.41 ± 2.17	30.98 ± 2.2	*N* = 8	Lipiski (51)
***Ovine***								
White Alpine, adult	3 years	70 ± 5 kg	2.85 ± 0.40				*N* = 4	Cabrera et al. (28)
Dorset, pediatric	6–15 wk	20.9 ± 2.2 kg				12.5–14	*N* = 4	Hofferberth et al. (15)
Dorset, adult		58.1 ± 2.7 kg				20–25	*N* = 4	Hofferberth et al. (15)
***Human***								
Pediatric	8 months		1.06 ± 0.36				*N* = 1 (7)	Cabrera et al. (28)
Pediatric	<6 months				17.9 ± 2.8		*N* = 22	Hofferberth et al. (15)
Pediatric	New born					8		Beekman et al. (17)
Pediatric, TOF	27.88 ± 28.27 months			10.44 ± 3.16				Shi (52)
Pediatric		BSA = 0.2 m^2^				8		Zilberman et al. (5)
Pediatric		BSA = 0.4 m^2^				12		Zilberman et al. (5)
Pediatric		BSA = 0.8 m^2^				16–17		Zilberman et al. (5)
Adult	40.9 ± 18.3 years		1.88 ± 0.38				*N* = 8	van den Abbeele et al. (38)
Adult	50 ± 12 years		1.5 ± 0.3				*N* = 21	Azadani (53)
Adult						26		Beekman et al. (17)

### Radial testing method

When designing medical implants to address patient-specific needs, the capability to assess an individual patient’s tissue properties non-destructively is desired, particularly for devices that impart force on the surrounding tissue. The novel radial testing method presented here demonstrated the ability to provide reasonably accurate tissue stiffness measurements for PA and PV tissue when compared to a traditional uniaxial technique. Uniquely, this was performed without altering or damaging the tissue region of interest. While this proof-of-concept study was performed on cadaveric hearts *ex vivo* and leveraged destructive alterations to improve ease of access, the long-term goal is to measure patient-specific properties *in situ.* With continued development, we plan to optimize the balloon catheter-based radial testing platform to directly test the implantation site of living preclinical models and patients. Ultimately, this technique could provide a real time analysis of patient tissue properties at the bedside and make patient-specific implant selection a reality.

Currently, the radial technique is limited by the amount and accuracy of data that it can collect. Determining the initial tissue dimensions for the strain calculations was challenging *ex vivo* in the intact heart. We acknowledge the uncertainty associated with estimating the PA inner diameter based on subtracting twice the wall thickness for the PA; however, given the current system set-up the internal diameter could not be measured directly during testing. Additionally, regarding the PV diameter measurements using axial imaging, an obscured balloon/tissue interface and difficulty maintaining a perfectly axial view may have caused an overestimation of balloon diameter, especially at lower pressures. This overestimation of balloon diameter likely resulted in an underreporting of strain values and may explain the discrepancy seen between the radial and uniaxial Young’s Modulus values for Yucatan PVs in the elastic region featured in Table 1 and Figure 4.

Pressure increase was used to assess the point at which tissue stress was initiated. However, filtering out the baseline pressure of the balloon as well as the force required to lift the tissue during balloon inflation added uncertainty to the determination of the zero stress and strain points. Additionally, *in situ* measurement techniques cannot completely isolate circumferential characteristics from longitudinal effects when applying radial force *in situ.* This is because the *ex vivo* tissue response to radial stretch has a greatly reduced contribution from longitudinal tension experienced *in vivo* since it has been cut out from the surrounding tissue. Finally, while tracking the expansion of the balloon can be performed using multiple techniques, measuring PA or PV wall thickness changes becomes difficult. We demonstrated that the error induced by using the thin wall assumption when calculating hoop stress and assuming negligible longitudinal effects resulted in comparable results to uniaxial testing, although it is possible that other approaches could improve agreement between methods. These remain areas for improvement of the radial technique in future work but do not detract from its overall promise to assess patient-specific tissue properties.

### Alignment of Young’s modulus with stress

The limited sample size, population diversity, and experimental limitations of this study resulted in relatively large variations in strain between and within our experimental groups (Figure 3). Across both breeds and tissue locations we observed large horizontal shifts in the stress response curves such that, for a given strain, individual samples span both the elastic and fibrous regimes. This may be attributed to multiple factors, such as experimental error in determining the appropriate r_0_ or l_0_ values, biological variations within the sample population’s homeostatic pressures, tissue thickness, PA or PV size, and tissue composition. Ultimately, a comparison of average stiffness for a given strain failed to provide an informative perspective of the population due to these large horizontal shifts between the curves. However, the error resulting from these shifts had a reduced effect on the calculated stiffness values, and thus improved alignment of the stress response curves was observed with respect to applied stress. Despite potential causes stemming from experimental limitations, the biological variations within the YU and YO piglet populations may play a large part in the strain variance observed. As such, it may be a reasonable hypothesis that since applied stress is more closely linked to the homeostatic pressure condition than strain, the tissue stress response is more closely linked to the applied pressure than its relative displacement from the unstressed condition. A curve fit approach was used to calculate Young’s modulus for a given stress (Figure 6). Using this method, good agreement between the radial and uniaxial measurements was observed, as there was no significant difference between methods at any given point. In addition, we used the Bland-Altman method to assess agreement between the new radial balloon catheter-based technique and the “gold standard” uniaxial testing method, similar to others (44). A limitation of the Bland-Altman method, however, is that it only defines limits of agreement and does not inform acceptability, which must be determined based on other requirements such as effects on clinical decisions (45). Our analysis showed that most of the measurements fell within the limits of agreement. Whether the limits are acceptable for future applications of the radial method remains to be determined, and we expect agreement to improve with further development of the technique.

Curve fit analysis aids in data visualization and comprehension by providing an analytic expression that enables the extraction of relevant tissue properties through the stress response curve. However, limitations such as regression overfitting can occur when pursuing a high coefficient of determination (R^2^) value in varying data. Overfitting can give rise to misrepresentation of the R^2^ and curve coefficients, consequently, expressing the noise instead of the full data set. Additionally, toward the ends of the dataset the need to extrapolate increases. This can cause complex fits to deviate from the available data and reduce local accuracy. Therefore, stiffness values for very low and high stress values may include a greater degree of regression artifacts.

### Limitations and challenges

This study has several limitations. Due to constraints in piglet availability and ethical considerations in minimizing the use of animals, sample sizes across breeds were small (YO *N* = 4–8, YU *N* = 4) and data was only collected from male piglets. Small tissue dimensions also limited the number of samples that could be taken from each heart: one from the PA and one from the PV. Not all measurements were performed on the YO population, and some had to be excluded. Only 4 YO piglets underwent *in vivo* measurements. One YO PA sample was tested with the radial method but had to be removed from the dataset due to early damage during uniaxial testing set-up. The radial technique was more challenging to apply to the PV tissue region than the PA, so the approach was still being optimized during YO PV testing. For this reason, this YO PV dataset was not included.

The range of data collected by the method of radial testing is limited by the balloon’s optimal diameter range. The balloon we used for this study had a fixed maximum diameter of 22 mm. As the maximum diameter is approached, the balloon material quickly becomes the dominant resistor to expansion. The control curve taken at the beginning of the radial testing procedure served to filter out any of the forces potentially imparted by the balloon at larger diameters. Furthermore, we noted at very low pressures (∼ < 5 mmHg) and low expansion (∼50%), folding of the balloon material resulted in inconsistent pressure distributions and slippage. However, this condition represents a very small area of our testing curves and does not account for significant error in the force ranges of interest. For these reasons, sub-maximal filling volumes are ideal as the balloon material provides little expansion resistance that is accounted for in our baseline subtraction. The balloon material is compliant such that at pressures > 5 mmHg it readily conforms to the tissue interface. We used a custom inflation pressure measurement system that accurately measured and adjusted pressure in the reported regions. Selecting a balloon for optimal measurements in the physiologic and near physiologic ranges resulted in attaining maximum working expansion before observing failure in the tissue. In order to collect data on the tissue’s ultimate tensile strength using the radial testing method, a follow-up study in which greater error in physiologic range is acceptable could be performed using a larger balloon.

There were minor changes made to the testing procedures between testing tissue samples from the YO and YU piglets. The YO cohort was tested first. After preliminary data analysis, it was decided that the pressure applied by the radial testing system should be reduced from 150 mmHg to 90 mmHg to prevent potential tissue damage prior to uniaxial testing. The stress induced by the radial apparatus during the preconditioning sequence was also increased from 50 mmHg to 90 mmHg to ensure hysteresis was reduced over the entire testing pressure range. The extension rate of the uniaxial apparatus was slowed down from 0.6 mm/s to 0.06 mm/s to match that of the radial testing apparatus, as extension rate can affect measured responses in viscoelastic tissues (46). It was assumed that the changes in measured responses as a result of this difference in extension rates would be negligible, but the change was made to the procedure as an extra precaution. The number of uniaxial preconditioning cycles was decreased from ten to five to reduce the total testing time. Before making this change, it was confirmed that the tissue response became consistent after five cycles. This rationale was also supported in literature (30, 47).

A direct comparison between the radial and uniaxial techniques to validate the accuracy of the resulting measurements is complex, as neither method can be confirmed to be unequivocally correct. We note that biaxial testing has become a preferred method used by many biomechanical groups. However, the uniaxial tensile test is well accepted across many fields, including studies of soft biological tissue. We chose uniaxial testing as our preferred method for comparison due to its straightforward process and alignment with the balloon method. In order to assess agreement, we used the Bland-Altman analysis method (32). As described above, a majority of the data for both techniques fell within the limits of agreement, although it remains to be determined what will be acceptable with improvement of the radial technique and future applications to the clinic.

Replicating the diameter measurements taken before excising the heart was challenging *ex vivo*. However, estimating the *in vivo* diameter of excised tissue is useful when access to the heart prior to removal is unavailable. The thawed tissue was unstressed and collapsed, thus making an accurate caliper measurement of diameter impossible. An attempt to restore the tissue to homeostatic pressure succeeded in re-establishing the physiologic shape but the static pressure resulted in overestimation of the diameter due to loss of active tissue properties. Measuring the circumference of the tissue after excision and unfurling provided the most reproducible results, albeit with the tissue in an unstressed condition. Additionally, the method of estimating the sample’s internal diameter by subtracting twice the average thickness from the external diameter was not ideal but proved to be accurate enough to align radial values with uniaxial values. Future studies could mitigate this limitation by using a more complex measuring system, such as x-ray and a contrast agent.

The irregular shape and non-uniform thickness of the PV region tissue samples made it more difficult to collect anatomical measurements and mechanical property data compared to the more uniform PA samples. The location of the PV annulus region is in the same plane as other complex anatomical features and prevents discrete isolation. In order to prepare samples consistently, cardiac structures (leaflets, adjacent valves, etc.) were used as guidelines, and multiple measurements were taken across the sample to account for variability in shape. Nevertheless, we acknowledge that these non-uniformities in the PV tissue, and to a lesser extent in the PA tissue, likely contributed to increased variability and potential error in our tissue stiffness calculations. Tissue non-uniformity also influenced our decision to cut samples into rectangles rather than the standard dog-bone shape for uniaxial testing, which was also supported by literature (26, 28).

Due to logistical constraints of the study, there was a delay between *in vivo* measurements and *ex vivo* testing; thus, tissue preservation was necessary. Based on various literature, freezing at −80°C was chosen as the preferred method for preserving bulk tissue mechanical properties (29, 31, 48, 49). The tissue was checked macroscopically for integrity but not microscopically, so it is possible that microscopic changes could have occurred and were not detected. Histological evaluation of the tissue in future work should be considered.

## Conclusion

The juvenile porcine PA and PV anatomical measurements and tissue stiffness data sets presented here fill an important gap in the available information for medical device designers planning pediatric device implantation studies in preclinical animal models. In terms of cardiac structures, juvenile YU and YO mini-swine may be suitable models to test pediatric cardiovascular prosthetics given similarity in PA and PV size and tissue stiffness to that of human infants and young children. Additionally, the radial testing technique presented here demonstrates the potential to provide non-destructive accurate tissue stiffness measurements compared against the well-established uniaxial testing method. With further development, the radial testing platform can be improved and transitioned into a clinical technique that can measure patient-specific tissue properties and enable the use of custom prostheses.

## Data availability statement

The original contributions presented in this study are included in the article/Supplementary material, further inquiries can be directed to the corresponding author/s.

## Ethics statement

The animal study was reviewed and approved by the Seattle Children’s Research Institute IACUC and the Department of Defense Animal Care and Use Review Office (ACURO).

## Author contributions

DS, AM, and CW developed the original idea for the study. MK and MP performed the *in vivo* animal measurements. DS, AM, and MA performed the *ex vivo* experiments and analyzed the data. GV, BI, and SS aided in design of the mechanical testing systems and experimental approaches. DS, AM, MA, GV, MK, and CW drafted the figures and manuscript with input from all authors. All authors contributed to the manuscript and approved the submitted version.
