# Supplementary material for: Characterization of main pulmonary artery and valve annulus region of piglets using echocardiography, uniaxial tensile testing, and a novel non-destructive technique
Source: Front Cardiovasc Med. 2022 Aug 26;9:884116. doi: 10.3389/fcvm.2022.884116 (PMC9459108; doi:10.3389/fcvm.2022.884116)
Supplement: Supplementary file 1 [file Data_Sheet_1.pdf]

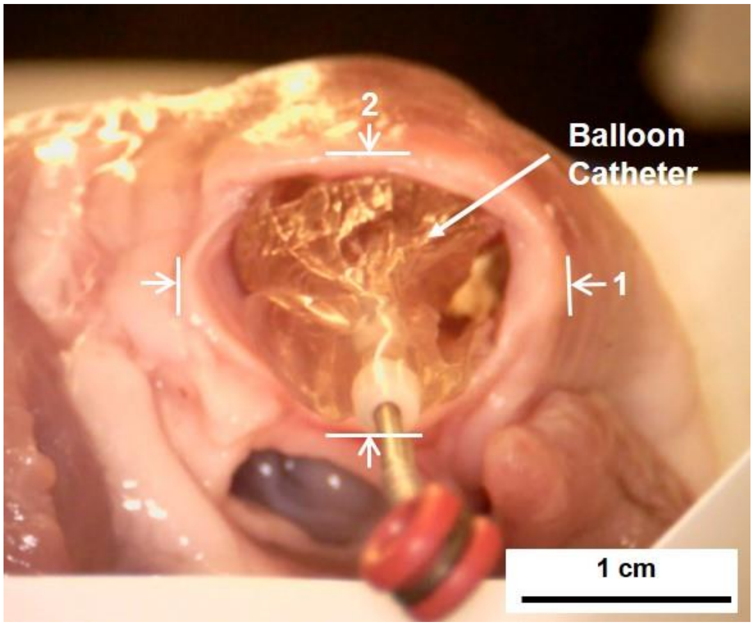

Supplementary Figure 1: Balloon expansion was recorded axially and PV diameter changes during testing were measured in two directions.

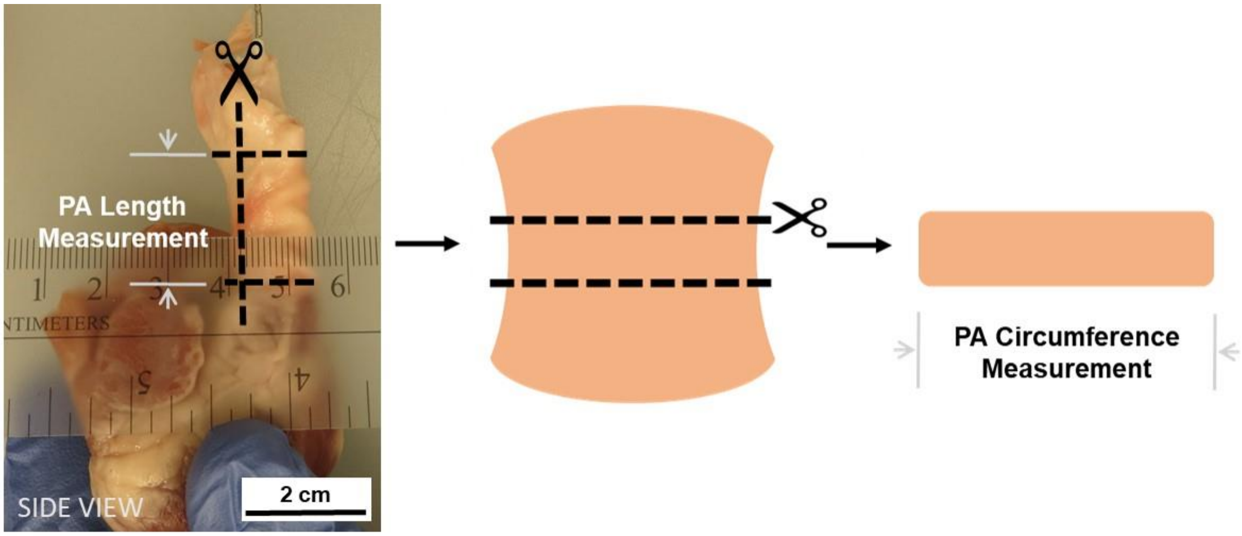

Supplementary Figure 2: The PA was isolated from the heart with 3 cuts. The first cut was horizontal to separate it from the rest of the heart, the second cut was horizontal to separate it from the bifurcation, and the third cut was vertical to unfurl it. The length of this cut was recorded as the PA length. A rectangular sample was cut from this unfurled tissue in the circumferential direction, the length of this sample was recorded as the PA circumference.

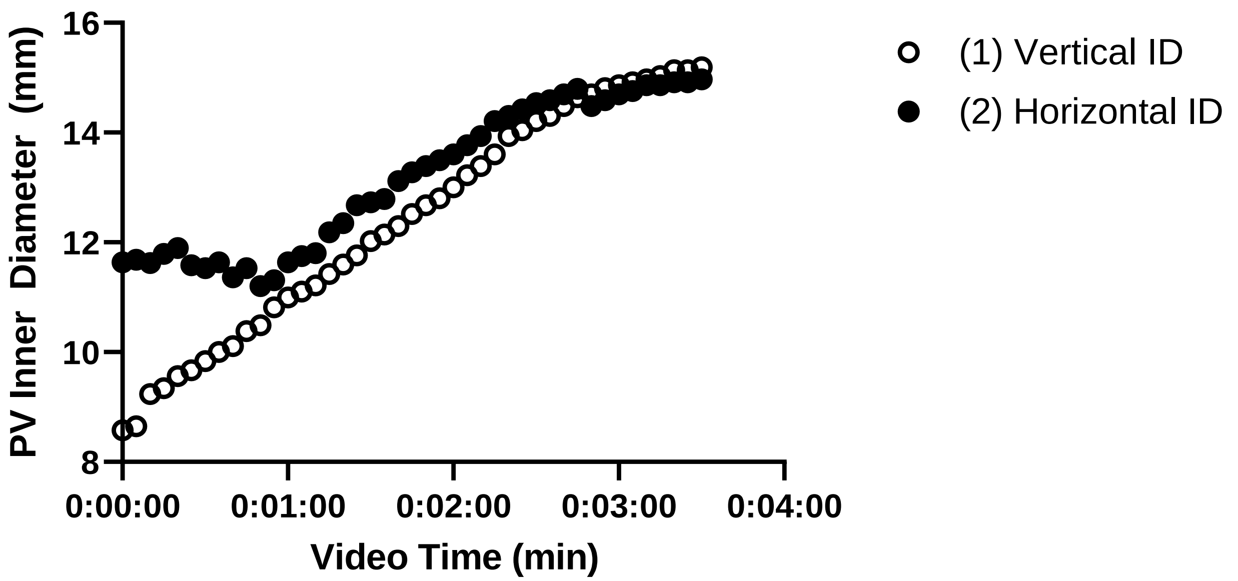

Supplementary Figure 3: During radial testing, a non-negligible amount of pressure was required to lift the PV tissue and restore it to a cylindrical shape. The inner diameter measured in the horizontal direction initially reduced as the inner diameter measured in the vertical direction increased until a constant relationship was reached and maintained for the remainder of the experiment.

Supplementary Table 1

Piglet Strain	Age (d)	Wt (kg)	LVP (mmHg)	AP (mmHg)	RVP (mmHg)	PAP (mmHg)	Caliper PA diameter (mm)	Echo PA diameter (mm)	Echo PV diameter (mm)
Yorkshire	41	8.9	74/6		25/6	27/19 (21)	12.94	12.3	9.2-10.9
Yorkshire	44	9.3	76/6		18/4	18/11 (13)	12.34	12.4-13.2	11.8
Yorkshire	40	9.3	66/7		15/5	15/11 (12)	13.12	10.7	9.1-11.7
Yorkshire	40	10.2	73/6		18/6	19/12 (14)	12.68	11.9-13.4	10.3-12.3
Yucatan	43	6		73/51 (58)	22/3	22/12 (15)	11	8.0-9.1	9.8
Yucatan	48	5.8		104/63 (76)	24/5	23/16 (18)	11	7.3-8.0	7.6
Yucatan	49	6.2		77/45 (55)	26/4	23/11 (15)	11.5	8.3-9.4	8.55
Yucatan	52	7.3		99/59 (72)	26/7	26/15 (18)	13	9.0-10.8	9.7

Supplementary Table 1: Tabulated age, weight (Wt), left ventricular pressure (LVP; systolic/end diastolic pressure), systemic arterial pressure (AP; systolic/diastolic (mean) pressure), right ventricular pressure (RVP; systolic/end diastolic pressure), pulmonary artery pressure (PAP systolic/diastolic (mean) pressure), PA diameter by caliper, PA diameter by Echo, and PV diameter by Echo. Note that all piglets used in the study were male.
